# Tumor-Derived Extracellular Vesicles Impair CD171-Specific CD4^+^ CAR T Cell Efficacy

**DOI:** 10.3389/fimmu.2020.00531

**Published:** 2020-03-31

**Authors:** Solin Ali, Karin Toews, Silke Schwiebert, Anika Klaus, Annika Winkler, Laura Grunewald, Lena Oevermann, Hedwig E. Deubzer, Alicia Tüns, Michael C. Jensen, Anton G. Henssen, Angelika Eggert, Johannes H. Schulte, Esther Schwich, Vera Rebmann, Alexander Schramm, Annette Künkele

**Affiliations:** ^1^Department of Pediatric Oncology and Hematology, Berlin Institute of Health, Charité—Universitätsmedizin Berlin, Corporate Member of Freie Universität Berlin, Humboldt—Universität zu Berlin, Berlin, Germany; ^2^Berlin Institute of Health (BIH), Berlin, Germany; ^3^Neuroblastoma Research Group, Experimental and Clinical Research Center (ECRC) of the Charité and the Max-Delbrück-Center for Molecular Medicine (MDC) in the Helmholtz Association, Berlin, Germany; ^4^German Cancer Consortium (DKTK), Heidelberg, Germany; ^5^German Cancer Research Center (DKFZ), Heidelberg, Germany; ^6^Department of Internal Medicine, University Duisburg-Essen, Essen, Germany; ^7^Ben Towne Center for Childhood Cancer Research, Seattle Children's Research Institute, Seattle, WA, United States; ^8^Fred Hutchinson Cancer Research Center, Seattle, WA, United States; ^9^University of Washington, Department of Bioengineering, Seattle, WA, United States; ^10^Department of Transfusion Medicine, University Duisburg-Essen, Essen, Germany

**Keywords:** immunotherapy, pediatric oncology, neuroblastoma, solid tumors, neurotrophic receptor tyrosine kinase

## Abstract

Chimeric antigen receptor (CAR) T cell efficacy against solid tumors is currently limited by several immune escape mechanisms, which may include tumor-derived extracellular vesicles. Advanced neuroblastoma is an aggressive childhood tumor without curative treatment options for most relapsed patients today. We here evaluated the role of tumor-derived extracellular vesicles on the efficacy of CAR T cells targeting the neuroblastoma-specific antigen, CD171. For this purpose, CAR T cell activation, cytokine production, exhaustion, and tumor cell-directed cytotoxicity upon co-culture was evaluated. Tumor-derived extracellular vesicles isolated from SH-SY5Y neuroblastoma cells neither affected CAR T cell activation nor expression of inhibitory markers. Importantly, exposure of CD4^+^ CD171-specific CAR T cells to tumor-derived extracellular vesicles significantly impaired tumor cytotoxicity of CAR T cells. This effect was independent of neurotrophic receptor tyrosine kinases 1 or 2 (NTRK1, NTRK2) expression, which is known to impact immune responses against neuroblastoma. Our results demonstrate for the first time the impact of tumor-derived extracellular vesicles and non-cell-mediated tumor-suppressive effects on CD4^+^ CAR T cell efficacy in a preclinical setting. We conclude that these factors should be considered for any CAR T cell-based therapy to make CAR T cell therapy successful against solid tumors.

## Introduction

Solid tumor therapy is faced with multiple obstacles that complicate efficient reduction, and ultimately, elimination of cancer cells. Immune surveillance and reinforcement of immune cell activity as a therapeutic option has become increasingly successful. The introduction of checkpoint inhibitors into clinical practice and the advent of cellular immunotherapy, such as CAR T cell-based therapy, holds great promise. Redirection of autologous T cells to a specific tumor-associated antigen via a transfected chimeric antigen receptor (CAR) creates augmented target specificity and improved T cell cytotoxicity. To date, the success of CAR T cell therapy has mostly been limited to the treatment of hematopoietic cancers (1–5). CAR T cell therapy for solid tumors can be hampered by an anti-inflammatory tumor microenvironment, which promotes immune escape mechanisms (6, 7). Here, intercellular communication plays a crucial role in establishing a pro-tumorigenic micromilieu that facilitates cancer spread and immune cell exclusion or dormancy (8). It is increasingly clear that communication between cells can be mediated over distant sites by secretion and uptake of extracellular vesicles, which carry multiple biologically active proteins on their surfaces and generally reflect the molecular composition of their parent cells. Tumor-derived extracellular vesicles (TEVs) are enriched in immunoregulatory proteins including FasL, PD-L1, inhibitory cytokines, classical, and non-classical MHC molecules and the corresponding tumor-associated antigens (9–12). PD-L1 on tumor exosomes is especially relevant in settings where patients are treated with antibodies against this checkpoint inhibitor. Importantly, exosomal PD-L1 levels correlated with response to therapy and suppression of exosomal PD-L1-induced systemic anti-tumor immunity (13, 14). These findings suggest that exosomal composition can massively impact immune cell-directed therapies.

Neuroblastoma, the most-common pediatric extracranial solid tumor, derives from neural precursor cells and displays a high biological as well as clinical heterogeneity (15, 16). Neuroblastoma ranges from disease that can spontaneously regress to high-risk cases for which patient survival is below 40% regardless of current standard multimodal therapy consisting of surgery, polychemotherapy, and radiation (15). Influential factors that can be of prognostic value are e.g., oncogene amplification, ploidy, allelic loss, and tumor stage. Furthermore, mutually exclusive expression of the neurotrophic receptor tyrosine kinases 1 or 2 (NTRK1, NTRK2) has prognostic significance, and both kinases contribute to distinct neuroblastoma biologies, in particular to differences in tumor immunogenicity (17). NTRK1 expression in neuroblastoma has been reported to be correlated with low tumor stage, enhanced DNA repair, a retention of immunogenicity associated with higher degree of leukocyte infiltration, a higher degree of differentiation, higher chemotherapeutic sensitivity, and an inhibitory effect on angiogenesis. All of these features conceivably contribute to excellent patient survival when tumors express NTRK1 (18–22). In contrast, NTRK2 expression in neuroblastoma has been reported to be associated with *MYCN* amplification, enhanced migratory properties leading to early metastases and a resistance to first-line chemotherapeutics, all of which contribute to poor patient survival (20, 22–24).

Previously, we preclinically evaluated CAR T cells targeting the CE7 epitope of the CD171 tumor-associated antigen in neuroblastoma models for their therapeutic efficacy as well as toxicity and safety (25, 26). CD171-directed CAR T cells are currently being tested in a phase I trial for patients with recurrent or refractory neuroblastoma (ClinicalTrials.gov Identifier: NCT02311621). Here we investigate the potential influence of TEVs on the efficacy of CD171-specific CAR T cells from CD4^+^ and CD8^+^ T cell subsets in preclinical neuroblastoma models and assess a potential differential involvement of neurotrophin receptors in this process.

## Materials and Methods

### Cell Culture

SH-SY5Y parental cells were maintained in RPMI Medium (Gibco) supplied with 10% fetal calf serum (FCS). Stable expression of NTRK1 or NTRK2 in SH-SY5Y human neuroblastoma cells was achieved as described before (27). SH-SY5Y-NTRK1 and SH-SY5Y-NTRK2 were cultivated in RPMI medium, supplied with 10% FCS and 500 μg/ml G418 (Sigma). All cell lines underwent Short Tandem Repeat DNA genotyping for cell line identification as well as weekly testing for mycoplasma using the PlasmoTest™ Kit (Invitrogen). The general number of passages between thawing and use was <20 for all experiments performed.

### Isolation of Extracellular Vesicles

To obtain extracellular vesicles released from SH-SY5Y, SH-SY5Y-NTRK1, and SH-SY5Y-NTRK2 cells, cells were cultured for 9 h in RPMI medium supplemented with 10% extracellular vesicle-depleted fetal bovine serum (FCS), 5% penicillin-streptomycin (Pen Strep, 10,000 U/mL, Life Technologies), and 1% L-glutamine (L-Glutamine, 200 mM, Life Technologies). Conditioned media was subjected to ultracentrifugation at 10,000 × g in a fixed angle Type 45 Ti rotor (Beckman Coulter) for 30 min in order to remove membrane patches, followed by a further ultracentrifugation step at 120,000 × g for 120 min at 4°C using a swinging bucket SW 40 Ti rotor (Beckman Coulter). Pelleted TEVs were resuspended in 0.9% NaCl and stored at −20°C until usage. The obtained TEV fractions were characterized by (i) SDS-PAGE and western blotting to verify typical extracellular vesicle marker expression (CD81, TSG101, syntenin) and the absence of intracellular proteins or endosomes (calnexin) according to consensus requirements defining extracellular vesicles (28), (ii) nano-particle tracking analysis using ZetaView analyses (Particle Metrix, Diessen, Germany) to define size and particle concentration (29) and (iii) protein assay (Thermo Scientific, Darmstadt, Germany) to define protein concentration.

### CAR Constructs

The CD171-specific CE7-CAR was cloned into the SIN epHIV7 lentiviral vector, and lentivirus was propagated in 293T cells (30, 31). The scFv was codon-optimized and subsequently linked to a 229-amino acid spacer domain from the human IgG4 hinge. The spacer domain was modified by two substitutions, L235D and N297Q, to reduce binding to the IgG Fc gamma receptor (32). The spacer domain connects the antigen-binding domain to the CD28 transmembrane domain, which is followed by the signaling module containing the CD3zeta cytoplasmic domain and 4-1BB. The CAR construct also contained a T2A self-cleaving peptide and truncated epidermal growth factor receptor (EGFRt) allowing for CAR T cell detection and enrichment.

### Generation and Cultivation of CD171-Specific CAR T Cells

Apheresis products were obtained from healthy donors (Charité ethics committee approval EA2/216/18) and peripheral blood mononuclear cells were isolated using Ficoll-Paque (GE Healthcare). CD4^+^ and CD8^+^ T cells were obtained by positive selection using immunomagnetic microbeads (Miltenyi Biotec), and activated with anti-CD3/CD28 beads (Life Technologies). On day three, activated T cells were transduced with the CAR-containing lentivirus. EGFRt^+^ CAR T cells were enriched by immunomagnetic selection with biotin-conjugated cetuximab (Bristol-Myers Squibb) and streptavidin microbeads (Miltenyi Biotec). Untransduced T cells were used as negative controls alongside CAR T cells in all experiments. CAR T cells and control T cells were cryopreserved until further use. Cryopreserved cells were thawed, stimulated with irradiated peripheral blood mononuclear cells, irradiated lymphoblastoid TMLCL cells, and OKT3 (30 ng/mL, Miltenyi Biotec), and expanded according to a rapid expansion protocol (26). CD4^+^ T cells were supplied with IL2 (50 U/μl) and IL7 (10 ng/μl) and CD8^+^ T cells were supplied with IL2 (50 U/μl) and IL15 (10 ng/μl) every other day following expansion. Functional *in vitro* assays were conducted between days 11 and 16 of culture.

### PCR

DNA was isolated from SH-SY5Y parental cells and the stable expressing SH-SY5Y-NTRK1 and SH-SY5Y-NTRK2 cell models using the Nucleospin Tissue Kit (Macherey-Nagel). PCR-based detection of neurotrophin receptor expression was achieved by PCR-based amplification using primers for NTRK1 (forward: ACCATGCTGCCCATTCGCTG, reverse: GAGGGCAGGCCCCAGTATTC) or NTRK2 (forward: GCAATGATGATGACTCTGCC, reverse: GGAACACTTTTCCAAAGGCT), and subsequent separation of PCR products in 1% agarose gels by electrophoresis.

### Western Blotting

Tumor cells were detached by trypsin, washed twice with PBS and lysed in RIPA buffer including protease inhibitors and the Phosphatase Inhibitor Cocktail (Roche). Proteins were separated by SDS-PAGE before western blotting with pan-Trk (C-14; sc-11, Santa Cruz Biotechnology) and phospho-Trk (#4621, Cell Signaling Technology) antibodies.

### Flow Cytometry

Cell-surface expression of CD4 (BD Biosciences), CD8 (BioLegend), and CD171 (cat#130-100-691, Miltenyi Biotec) was detected by fluorophore-conjugated monoclonal antibodies. EGFRt expression was detected using biotinylated cetuximab (Bristol-Myers Squibb) and a phycoerythrin (PE)-conjugated streptavidin antibody (BioLegend). T cell activation and exhaustion were assessed by fluorophore-conjugated monoclonal antibodies detecting CD137 (BioLegend), CD25 (BioLegend), PD-1 (also known as PDCD1 or CD279, BioLegend), TIM3 (Biolegend), and LAG3 (BD Biosciences). Flow cytometry was performed on a Fortessa X-20 (BD Biosciences) and data were processed using FlowJo software (Tree Star Inc.). Dead cells were excluded from analyses using LIVE/DEAD^TM^ Fixable Green Dead Cell Stain Kit (Life Technologies).

### TEV Co-culture and Exposure

T cells (2 × 10^5^) were co-cultured with 10 μg TEV protein per well in 96-well flat-bottom plates in triplicate for 24 h in RPMI supplemented with 10% extracellular vesicle-depleted FCS. After 24 h cells were pooled, washed twice with PBS and either used in FACS analyses or for viability assessment using trypan blue. T cells were then mixed with tumor cells at an effector:target ratio of 1:2. T cells primed with TEVs derived from parental SH-SY5Y cells were co-cultured with SH-SY5Y target cells. T cells primed with TEVs derived from SH-SY5Y-NTRK1 or SH-SY5Y-NTRK2 cells were co-cultured with SH-SY5Y-NTRK1 or SH-SY5Y-NTRK2 target cells, respectively. Viability, activation and exhaustion were assessed in CAR T cells after 24 h using FACS-based detection of cell surface markers.

### Functional Assays

For cytokine release assays, 2 × 10^5^ T cells were seeded together with stimulator cells at a 1:2 effector:target ratio. After 24 h, conditioned media was collected and stored at −80°C until analysis of IL2 and IFNG using the OptEIA™ ELISA (BD Biosciences) according to the manufacturer's instructions. CAR T cell-induced cytotoxicity was quantified in a biophotonic luciferase assay in which the neuroblastoma cells stably transduced with the GFP-ffLuc_epHIV7 reporter plasmid served as tumor target cells. Target cells were co-cultured with negative control or CAR T cells. The maximal biophotonic luciferase signal was obtained by measuring luminescence of target cells in the absence of CAR T cells (RLU_max_, maximal relative light unit). After 24 or 72 h, 0.14 mg D-luciferin (PerkinElmer Inc.)/ml medium was added to each well, and the biophotonic signal was detected. Lysis was determined as [1-(RLU_sample_/RLU_max_)] × 100 in relation to untreated cells. For sequential treatment, the additive amount of tumor lysis was calculated related to viable tumor cells at day 3. If not indicated otherwise, all data points were obtained as technical triplicates.

### Statistical Analysis

The differences between treatment groups were statistically analyzed using unpaired Student *t* tests in PRISM (GraphPad Software, Inc., San Diego, CA, USA). *P* < 0.05 were considered statistically significant.

## Results

### Neuroblastoma TEVs Do Not Impair CAR T Cell Viability

**Figure 1:**
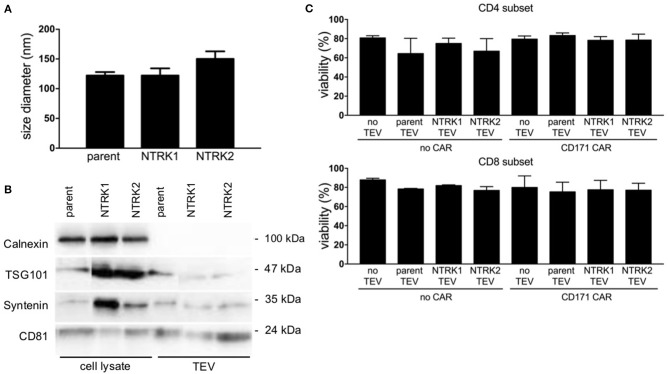
Characterization of extracellular vesicles preparations derived from SH-SY5Y and SH-SY5Y-NTRK cells by Nanotracking analysis and immunoblotting. **(A)** The mean TEV size did not vary among the different TEV preparations (*n* = 9). **(B)** Immunoblotting of TEV preparation and cell lysates for typical TEV markers TSG101, Syntenin, and CD81. Calnexin as protein of endoplasmic reticulum served as negative control. **(C)** CD4^+^ and CD8^+^ T cell viability after 24 h co-culture with 10 μg extracellular vesicles, accessed via flow cytometry after live-dead staining. Depicted is mean of three independent experiments with each using technical triplicates with error bars representing standard deviation (SD).

Since TEVs have been reported to modify immune effector functions (33, 34), we investigated the impact of extracellular vesicles derived from the human SH-SY5Y neuroblastoma cell line, which is lacking neurotrophin receptor expression, or neurotrophin-expressing models on CAR T cell efficacy. Models stably expressing each neurotrophin receptor were used to explore the impact of neurotrophin receptor expression on TEVs and CD171-targeting CAR T cell efficacy *in vitro*. Ectopic NTRK1 and NTRK2 expression in SH-SY5Y cell models (referred to hereafter as SH-SY5Y-NTRK1 and SH-SY5Y-NTRK2, respectively) was validated using RT-PCR and western blotting. Constitutive activation was verified for both receptors as previously reported (Supplementary Figure 1) (35). TEVs were isolated from medium conditioned by SH-SY5Y, SH-SY5Y-NTRK1, or SH-SY5Y-NTRK2 cells. Nanoparticle tracking-based characterization of the TEV preparation revealed that vesicle diameter ranged between 120 and 150 nm, which is typical for extracellular vesicles (for further details see Supplementary Table 1). The CD81 tetraspanin protein and the syntenin and TSG101 cytosolic proteins, which are either ESCRT complex-associated proteins or linked to vesicle release, were detected in all extracellular vesicles preparations, but the intracellular protein, calnexin, was not (Figures 1A,B). This marker profile confirmed TEV preparation purity, and excluded contamination with cellular debris. To investigate the impact of extracellular vesicles derived from SH-SY5Y parental or NTRK-expressing cells on CAR T cell viability, we co-incubated CAR T cells with 10 μg TEV, then subsequently assessed viability. Viability of CD4^+^ or CD8^+^ CAR T or control T cells was not significantly altered by 24 h of co-culture with extracellular vesicles derived from SH-SY5Y parental or NTRK-expressing cells (Figure 1C).

### TEV Priming Impairs CD4^+^ CAR T Cell Cytotoxicity, While CD8^+^ CAR T Cell Activity Is Unaffected

**Figure 2:**
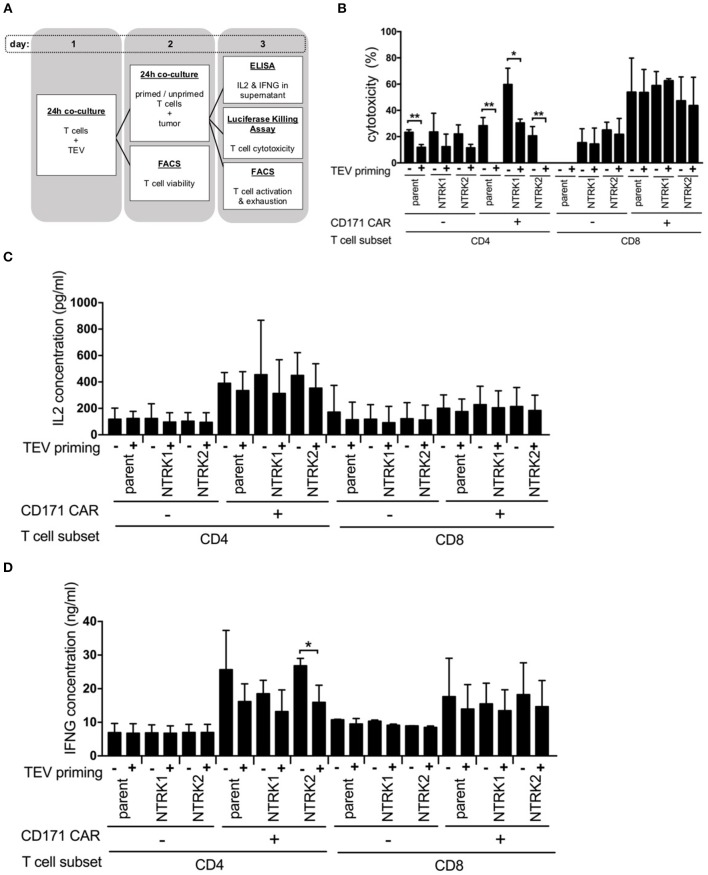
TEV-priming results in impaired CD4^+^ CAR T cell cytotoxicity. **(A)** Illustration of co-culture experiments with prior TEV-priming of CAR T cells. Co-culture experiments were conducted in three independent experiments with technical triplicates each. **(B)** Cytotoxicity assay of TEV-primed and unprimed CAR T cells measured biophotonically after co-incubation with GFP-ffluc expressing NTRK1 or NTRK2 expressing SH-SY5Y cell lines for 24 h at an effector:target ratio of 1:2. Co-culture experiments were conducted in three independent experiments with technical triplicates each. **(C)** Concentration of IL2 and **(D)** IFNG from supernatant of co-culture of TEV-primed and unprimed CAR T cells with tumor cells at an effector:target ratio of 1:2 quantified by ELISA. Cytokine measurements were conducted in three independent experiments with technical triplicates each. All experiments are depicted with error bars representing SD. *p < 0.05; **p < 0.01.

Next, we explored whether TEVs could influence CAR T cell efficacy. CAR T cells were exposed to TEVs for 24 h before co-incubation with tumor cells and assessment of cytotoxicity (Figure 2A). For this purpose, SH-SY5Y, SH-SY5Y-NTRK1, and SH-SY5Y-NTRK2 cells were transduced with a GFP-firefly luciferase reporter plasmid to facilitate viable tumor cell quantification. Cytotoxicity was assessed following 24 h of co-culture measuring residual luminescence signals of viable tumor cells. TEV priming of CD4^+^ CAR or control T cells significantly impaired the killing activity of these cells, regardless of neurotrophin receptor expression in the SH-SY5Y target cells, whereas the TEV priming of CD8^+^ CAR or control T cells did not affect neuroblastoma cell cytotoxicity (Figure 2B). Control T cells demonstrated a comparably low degree of cytotoxicity, most likely due to alloreactivity. Co-culturing TEV-primed CD171-specific CD4^+^ or CD8^+^ CAR T cells with SH-SY5Y or SH-SY5Y-NTRK cells consistently reduced the release of IL2 or IFNG by either CAR T cell type compared to unprimed T cells, although comparisons only reached significance for the CD4^+^ CAR T cells co-incubated with SH-SY5Y-NTRK2 cells (Figures 2C,D). FACS analyses revealed no differences in expression of activation markers or inhibitory receptors on primed vs. unprimed CD171-specific CD4^+^ and CD8^+^ CAR T cells after tumor cell co-culture (Supplementary Figures 2A,B). Our data demonstrate that TEV priming reduces CD171-specific CD4^+^ CAR T cell efficacy, but does not affect CD8^+^ CAR T cell-mediated cytotoxicity.

### NTRK1 Expression Augments CAR T Cell Cytotoxicity

**Figure 3:**
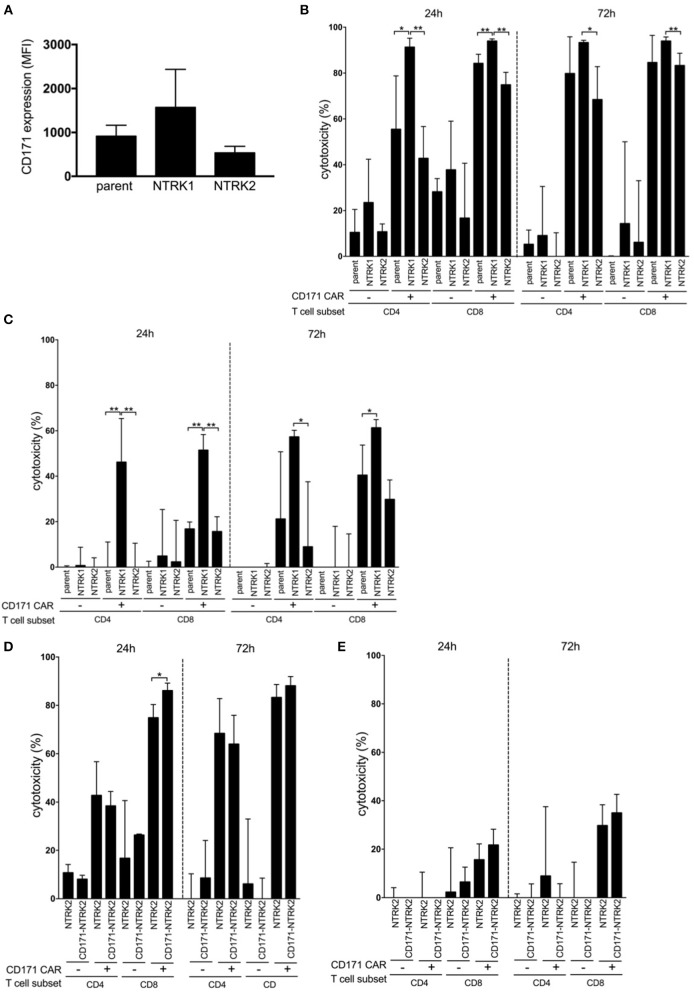
SH-SY5Y-NTRK1 increases CD171 expression in tumor cell lines and leads to improved CAR T cell cytotoxicity that is not significantly dictated through CD171 expression on the target tumor cell line. **(A)** Mean CD171 surface expression intensity (MFI) on SH-SY5Y-NTRK1, SH-SY5Y-NTRK2, and parental control by flow-cytometric analysis. Assessment of CD171 surface expression was done in three independent experiments. Expressed in mean with error bars representing SD. **(B)** Luciferase-based cytotoxicity assay of CD171-specific CAR T cells from CD4^+^ or CD8^+^ subsets against GFP-ffluc expressing SH-SY5Y-NTRK1, -NTRK2 and parental control measured after 24 and 72 h in an effector:target ratio of 2:1 and **(C)** 1:2. **(D)** Luciferase-based cytotoxicity assay of CD171-specific CAR T cells from CD4^+^ or CD8^+^ subsets against CD171-high SH-SY5Y-NTRK2 measured after 24 and 72 h in an effector:target ratio of 2:1 and **(E)** 1:2. Cytotoxicity assays were conducted in four independent experiments with technical triplicates each and expressed in mean with error bars representing SD. *p < 0.05; **p < 0.01.

Since neurotrophin receptor expression on neuroblastoma cells is known to have an effect on immune cells, we investigated whether NTRK1 or NTRK2 expression influences CAR T cell efficacy. We first determined CD171 expression in SH-SY5Y and SH-SY5Y-NTRK cells using flow cytometry. Interestingly, CD171 expression was modulated by NTRK receptor expression (Figure 3A), and was highest in NTRK1-expressing SH-SY5Y cells. We next examined whether the level of target-antigen expression on tumor cells altered CAR T cell function. CD171-specific CAR T cells were co-cultured with neuroblastoma reporter cells at an effector:target ratio of 2:1 or 1:2, respectively. Cytotoxicity was assessed following 24 and 72 h of co-culture measuring residual luminescence signals of viable tumor cells. At an effector:target ratio of 2:1, neuroblastoma cell killing was significantly more effective in the presence of CD4^+^ and CD8^+^ CD171-specific CAR T compared to control T cells (Figure 3B). After 24 h, CD4^+^ CAR T cell-directed SH-SY5Y-NTRK1 cytotoxicity was significantly higher than cytotoxicity to SH-SY5Y control cells (*p* = 0.023) or SH-SY5Y-NTRK2 cells (*p* = 0.0005). CD8^+^ CAR T cells mediated cytotoxicity even more strongly compared to CD4^+^ CAR T cells, and killing efficacy was significantly higher toward SH-SY5Y-NTRK1 compared to parental control cells (*p* = 0.005) or SH-SY5Y-NTRK2 cells (*p* = 0.0005, mean 94% vs. 84.2% vs. 74.9% after 24 h). These differences in killing efficacy were more prominent at effector:target ratios of 1:2. In contrast, control T cells displayed moderate or no cytotoxicity at effector:target ratios of 2:1 and 1:2, respectively (Figures 3B,C). In order to assess whether cytotoxicity induced by CD171-specific CAR T cells depended on antigen expression levels, we engineered SH-SY5Y-NTRK2 cells, which express only low CD171 levels, to over-express CD171 (Supplementary Figure 3). Cytotoxicity of CD4^+^ CAR T cells (effector:target ratio of 2:1) was not significantly affected by CD171 expression on SH-SY5Y-NTRK2 cells after 24 h (mean 42.8% vs. 38.4%) or 72 h (mean 68.4% vs. 64%, Figure 3D). Control T cells displayed little or no cytotoxicity against target cells, regardless of CD171 expression level. CD8^+^ CAR T cells displayed higher cytotoxicity against both cell lines when compared to CD4^+^ CAR T cells, reaching a maximum killing efficiency at an effector:target ratio of 2:1 when CD171 expression was high. At lower effector:target ratios (1:2), tumor cell killing was decreased regardless of CD171 expression levels in SH-SY5Y-NTRK2 cells (Figure 3E). Again, we observed comparably low and unspecific alloreactive cytotoxicity of the control T cells. These experiments demonstrate that differential NTRK expression on neuroblastoma cells alters CD171-directed CAR T cell cytotoxicity, and that this effect is independent of endogenous CD171 expression levels on target cells.

### NTRK1 Induces Elevated Expression of Activation Markers in CAR T Cells

**Figure 4:**
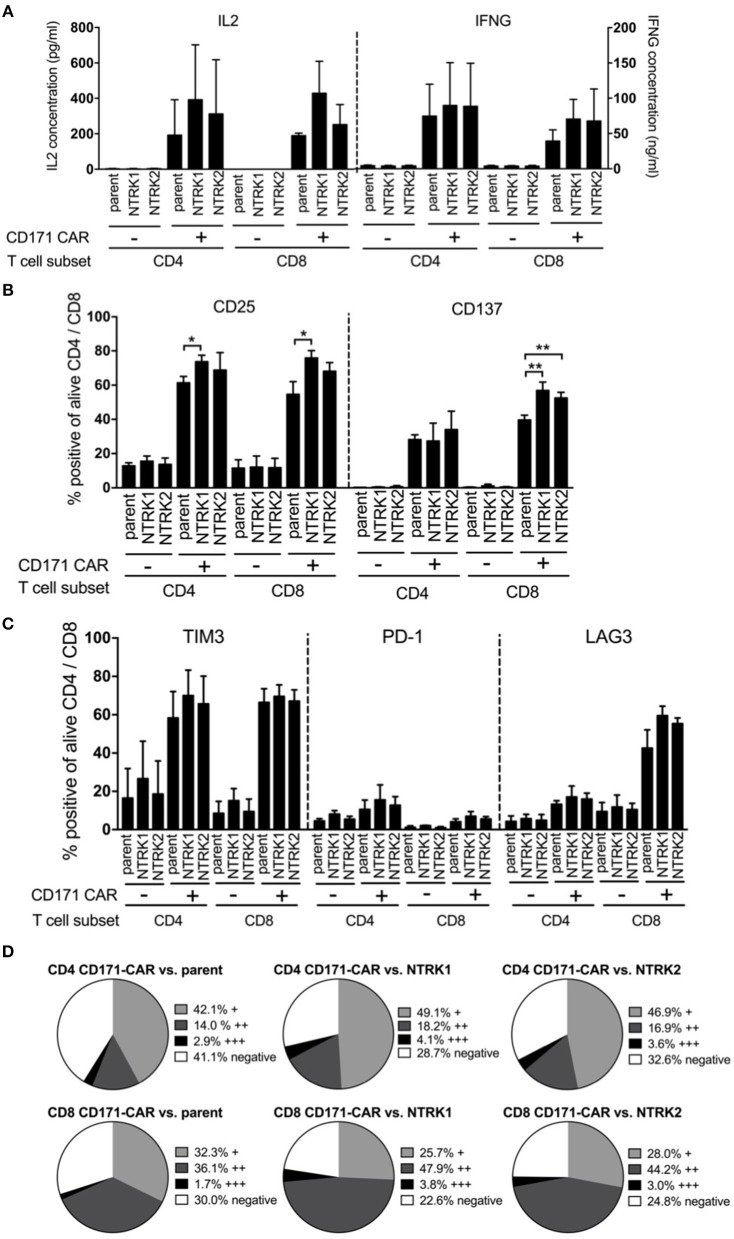
SH-SY5Y-NTRK1 expression results in increased CAR T cell activity, whereas NTRK has no significant influence on cytokine production and expression of exhaustion markers in CAR T cells. **(A)** Concentration of cytokines IL2 and IFNG in supernatant of CAR T cell and tumor in an effector:target ratio of 1:2 after 24 h co-culture determined by sandwich ELISA. Flow cytometric analysis of T cell surface activation markers CD25 and CD137 on CAR and control T cells after 24 h tumor co-culture. Depicted are double positive CAR and control T cells for CD4^+^ or CD8^+^ and CD25 or CD137. Cells were gated from living single cells. **(B)** Flow cytometric analysis of surface activation markers: Proportion of CD25 and CD137 on CAR T cells after tumor co-culture for 24 h in an effector:target ratio of 1:1 are shown. Depicted are double-positive cells for CD4^+^ or CD8^+^ and CD25 or CD137. Cells were gated from living single cells. Flow cytometric analysis was conducted in three independent experiments. **(C)** Flow cytometric analysis of surface exhaustion markers: Proportion of TIM3, PD-1, and LAG3 on CAR T cells after tumor co-culture for 24 h in an effector:target ratio of 1:1. Depicted are double-positive cells for CD4^+^ or CD8^+^ and TIM3, PD-1, or LAG3. Cells were gated from living single cells. Flow cytometric analysis was conducted in three independent experiments. **(D)** Distribution of no, single (+), double (++), or triple (+ + +) expression of exhaustion markers on CD4^+^ and CD8^+^ CAR T cells after 24 h tumor co-culture in an effector:target ratio of 1:1. Single positivity (+) was defined as the sum of TIM3^+^/PD-1^−^/LAG3^−^, TIM3^−^/PD-1^+^/LAG3^−^ populations and TIM3^−^/PD-1^−^/LAG3^+^ populations. Double positivity (++) was defined as the sum of TIM3^+^/PD-1^−^/LAG3^+^ populations, TIM3^+^/PD-1^+^/LAG3^−^ populations and TIM3^−^/PD-1^+^/LAG3^+^ populations. Triple positivity (+ + +) was defined as TIM3^+^/PD-1^+^/LAG3^+^ populations. Negative expression was defined as TIM3^−^/PD-1^−^/LAG3^−^ populations. Bars showed mean values with error bars representing SD. *p < 0.05; **p < 0.01.

NTRK-induced modulation of CAR T cell cytotoxicity was further analyzed using *in vitro* assays. Cytokine release (IL2 and IFNG) from CAR T cells following co-culture with SH-SY5Y, SH-SY5Y-NTRK1, and SH-SY5Y-NTRK2 cells was quantified by ELISA. Co-incubation with either the parental SH-SY5Y cell line or cell models expressing a neurotrophin receptor induced IL2 and IFNG release from CD171-specific CAR-T cells, but not control T cells (Figure 4A). Activation markers (CD25 and CD137) on CD4^+^ and CD8^+^ T cells harboring the CD171-specific CAR were induced when co-cultivated with SH-SY5Y cells, and this activation was independent of NTRK receptor expression (Figure 4B). CD25 upregulation on CD4^+^ and CD8^+^ CAR T cells was significantly more pronounced in the presence of SH-SY5Y-NTRK1 compared to parental control cells. Co-cultivation with either SH-SY5Y-NTRK1 or SH-SY5Y-NTRK2 (compared to parental control cells) significantly increased CD137 expression on CD8^+^ CAR T cells. In contrast, co-incubation with different neuroblastoma cells yielded mixed results on the expression of PD-1, TIM3 and LAG3 inhibitory receptors on CAR T cells. While TIM3 expression was significantly induced by exposure to either SH-SY5Y cells or the NTRK-expressing models, PD-1 expression on CD171-directed CAR T cells remained low in all co-culturing conditions analyzed (Figure 4C). Induction of LAG3 expression was restricted to CD8^+^ CD171-directed CAR T cells, independent of NTRK expression on target cells. Potential shifts in the distribution of the inhibitory receptor repertoire on CAR T cell subpopulations after exposure to SH-SY5Y cells and the NTRK-expressing derivates were investigated using pie charts visualizing the degree of inhibitory receptor expression in CAR T (Figure 4D) and control T cells (Supplementary Figure 4). While relative changes in CD4^+^ and CD8^+^ CAR T cell subpopulations expressing single, double or all three inhibitory receptors were highest after co-culture with SH-SY5Y-NTRK1 and lowest after co-culture with the parental cell control, these effects did not reach statistical significance. Our results demonstrate that co-cultivation with SH-SY5Y cells induced cytokine release, up-regulated different activation markers as well as the inhibitory LAG3 and TIM3 markers, but not PD-1, on CD171-specific CAR T cells.

## Discussion

Cell-based immunotherapies such as CAR T cells are now entering clinical testing for solid tumor treatment. Although the mechanism of action has been thoroughly investigated in childhood tumors including neuroblastoma (26, 36), little is known about host factors that could potentially modify therapeutic efficacy. TEVs have repeatedly shown to be key players in solid tumor formation and maintenance as well as immunosurveillance (13, 14). Here we analyzed the interplay between extracellular vesicles from preclinical neuroblastoma models with differential expression of the master regulators, NTRK1 and NTRK2, and CAR T cells, in order to identify molecular factors that can be investigated and maybe even manipulated to improve response to CAR T cell therapy. While CAR T cell viability, expression of activation and exhaustion markers as well as their cytokine production was largely unaffected, CD4^+^ CAR T cell cytotoxicity was significantly reduced in the presence of neuroblastoma-derived extracellular vesicles independent of NTRK1/2 expression. These results are in line with previous reports highlighting CD4^+^ T cell sensitivity toward exosomes and their suppressive potential (37, 38). However, we did not observe decreased T cell viability in the presence of TEVs that was described for TEVs derived from other tumor types (37, 39). Taken together these results suggest that suppressive effects of TEVs on T cell viability could vary depending on the tumor entity from which they are derived and on different CAR T cell subsets.

Even though NTRK1 and NTRK2 share extensive sequence similarity and similar proximal signaling targets, their expression is correlated with divergent effects on neuroblastoma biology and malignancy (27, 35). NTRK1 and NTRK2 expression are indicative of excellent and poor clinical outcome in neuroblastoma, respectively, yet there is no data on the role of NTRKs in cell-cell communication mediated by extracellular vesicles. We show that expression of the NTRK1 neurotrophin receptor in neuroblastoma cells augmented CAR T cell cytotoxicity and activation compared to cells not expressing NTRK1. In contrast, NTRK2 expression did not affect CAR T cell cytotoxicity. This effect could not be attributed to altered CAR T cell cytokine production, which was similarly induced upon co-incubation with tumor cells, irrespective of NTRK1 expression. These results are in line with previously published reports that NTRK1 expression on neuroblastoma cells enhanced proliferation, activation and IFNG production in healthy donor T cells (21, 27). These and other mechanisms, including MHC class I re-expression in neuroblastoma cells stably transfected to express NTRK1 and upregulation of immune modulating genes by NTRK1, corroborate the idea that excellent prognosis in patients with NTRK1-expressing neuroblastomas could be attributed at least in part through pro-immunogenic features mediated by NTRK1 (21).

It has been reported that TEVs reprogram CD8^+^ T cells to become suppressor cells inhibiting proliferation of other T cells (40). TEVs also inhibit T cell proliferation and cytolytic activity in effector T cells and other effector immune cells such as natural killer cells, and promote regulatory T cell proliferation and formation of myeloid-derived suppressor cells through their immunosuppressive cargo (9, 40–42). Of note, cytotoxic activity of CD171-specific CAR T cells was independent of NTRK2 expression, which is a marker of aggressive neuroblastoma. It remains to be elucidated whether NTRK2 inhibition by small molecules, which is highly effective against preclinical neuroblastoma models (23, 43), would further improve CAR T cell therapy against NTRK2-expressing neuroblastomas. Single agent-targeting of NTRK2, such as larotrectinib for NTRK2-rearranged tumors in adults and children (44), has now been approved for clinical application. Since TEVs, regardless of their derivation from NTRK1- or NTRK2-expressing cells, display obstructing effects on CD4^+^ CAR T cells *in vitro*, it should be tested whether CD8^+^ CAR T cells are a better suited for cell-based neuroblastoma immunotherapy. Vice versa, Tang et al. showed that CAR T cell-derived extracellular vesicles had potent anti-tumor properties and preserved their tumor-targeting specificity (45). Thus, it is likely that the interplay of tumor-derived and CAR T cell-derived extracellular vesicles reprograms the tumor microenvironment and needs to be considered when optimizing CAR T cell therapy for solid tumors.

A limitation of this study is the use of only one cell line. Nevertheless, the neuroblastoma cell line SH-SY5Y ± NTRK1/2 expression is a well-accepted and commonly used model for neuroblastoma allowing in-depth analysis.

Here we demonstrate that CD171-specific CAR T cells are functional and cytotoxic in preclinical models of neuroblastoma with different neurotrophin receptor expression, thus, covering a wide range of neuroblastoma biology. NTRK1-expressing neuroblastoma cells were most susceptible to both CD4^+^ and CD8^+^ CAR T cell-mediated killing, while NTRK2-expressing cells were significantly more resistant. TEVs abrogated the effect of CD4^+^, but not CD8^+^, CAR T cells independent of whether the tumor cells from which they were derived expressed NTRK receptors. Our results demonstrate that non-cell-mediated tumor-suppressive effects must be taken into account for any CAR T cell-based therapy.

## Data Availability Statement

The datasets generated for this study are available on request to the corresponding author.

## Ethics Statement

The studies involving human participants were reviewed and approved by Charité ethics committee approval EA2/216/18. The patients/participants provided their written informed consent to participate in this study.

## Author Contributions

SA performed the experiments. SA, AS, VR, and AKü were major contributors to study concepts, study design, and manuscript writing. MJ provided the CD171-specific CE7-CAR construct. SA, AKü, KT, AKl, SS, AW, LG, LO, HD, and AH contributed to conduction of experiments, analysis of data, quality control, and interpretation of data. AT and AS provided the NRTK1 and NTRK2 expressing cell lines. VR and ES provided the tumor-derived extracellular vesicles. AE and JS were involved in revising the article for important intellectual content. All authors approved the final manuscript.

### Conflict of Interest

The authors declare that the research was conducted in the absence of any commercial or financial relationships that could be construed as a potential conflict of interest.
