# Supplementary material for: Tumor-Derived Extracellular Vesicles Impair CD171-Specific CD4+ CAR T Cell Efficacy
Source: Front Immunol. 2020 Mar 31;11:531. doi: 10.3389/fimmu.2020.00531 (PMC7137471; doi:10.3389/fimmu.2020.00531)
Supplement: Supplementary file 1 [file Data_Sheet_1.docx]

Supplementary Material

# Supplementary Table 1

Overview of the different extracellular vesicle preparations and their corresponding characteristics regarding particle, protein concentration, purity and size.

| **EV** | **Particle**  **[/mL]** | **Protein**  **[mg/mL]** | **Purity**  **[particle/mg protein]** | **Mean Particle size**  **[nm]** |
| --- | --- | --- | --- | --- |
| SH-SY5Y | 7.1 x10e10 | 6.1 | 1.2 x10e10 | 122 |
| SH-SY5Y-NTRK1 | 3.8 x10e10 | 5.9 | 0.6 x10e10 | 122 |
| SH-SY5Y-NTRK2 | 1.9 x10e10 | 4.2 | 0.5 x10e10 | 150 |

# Supplementary Figures

#
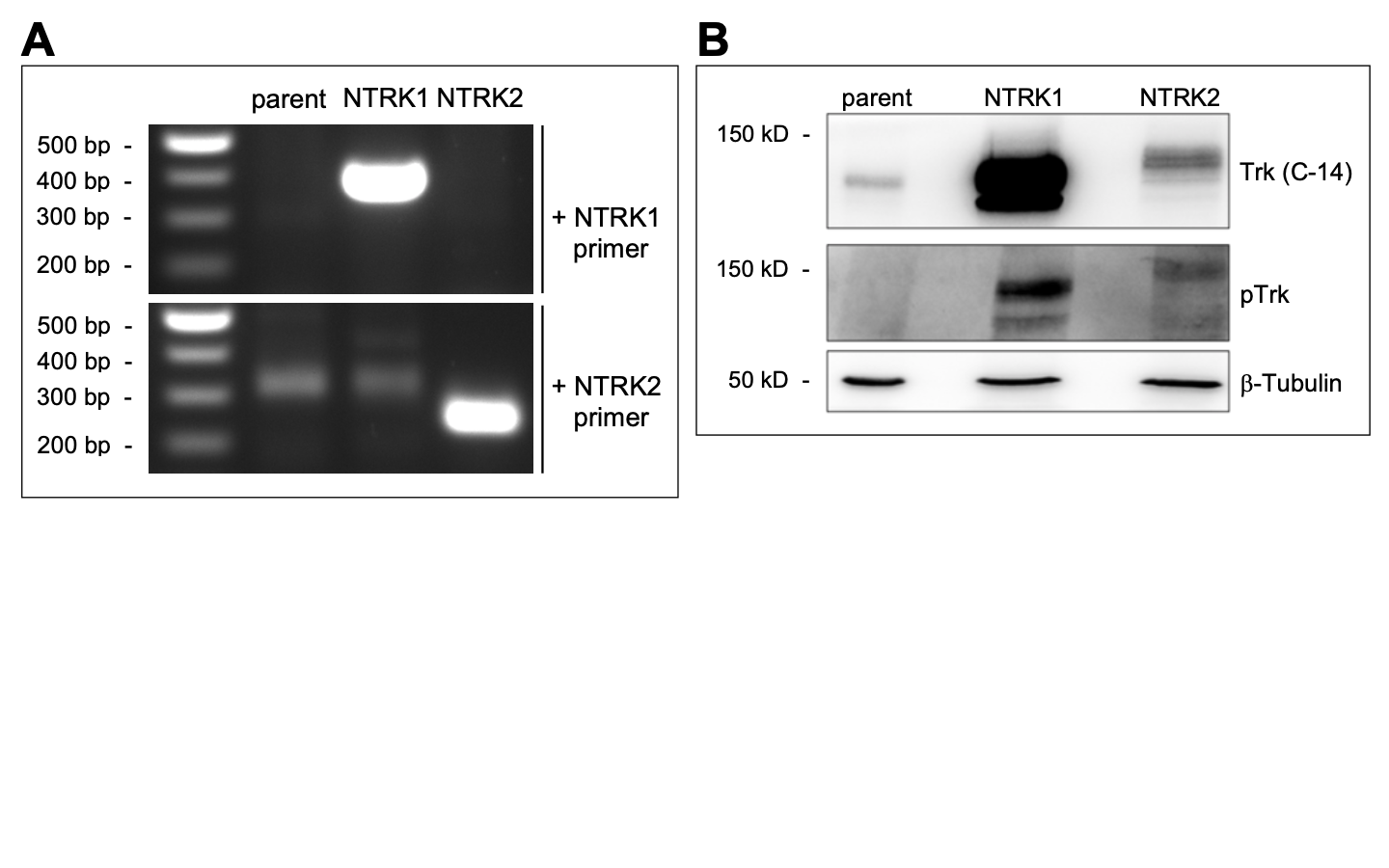
 Supplementary Figure 1. SH-SY5Y model successfully expresses NTRK1 or NTRK2 constructs at genomic level and display activation at proteomic level. (A) PCR displaying the presence of NTRK1 or NTRK2 receptors with TrkA and TrkB specific primers in the transfected SH-SY5Y cell lines. Bands depict NTRK1 cDNA at 386 bp and NTRK2 cDNA at 246 bp. (B) Western blot analysis of Trk (C-14), phosphorylated Trk (pTrk) and β-Tubulin as loading control. 30µg protein from each cell line were loaded into each lane.

##
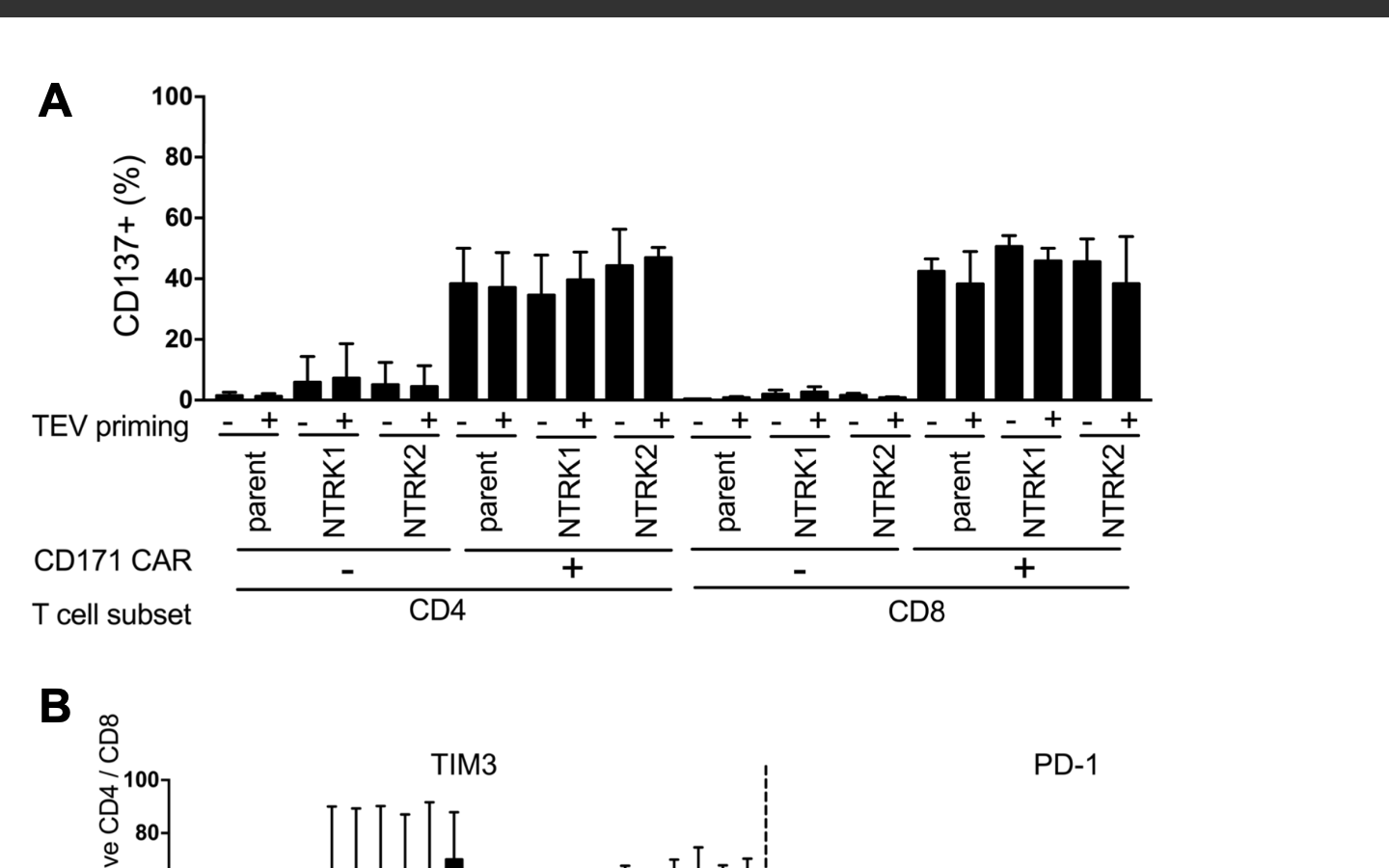


##
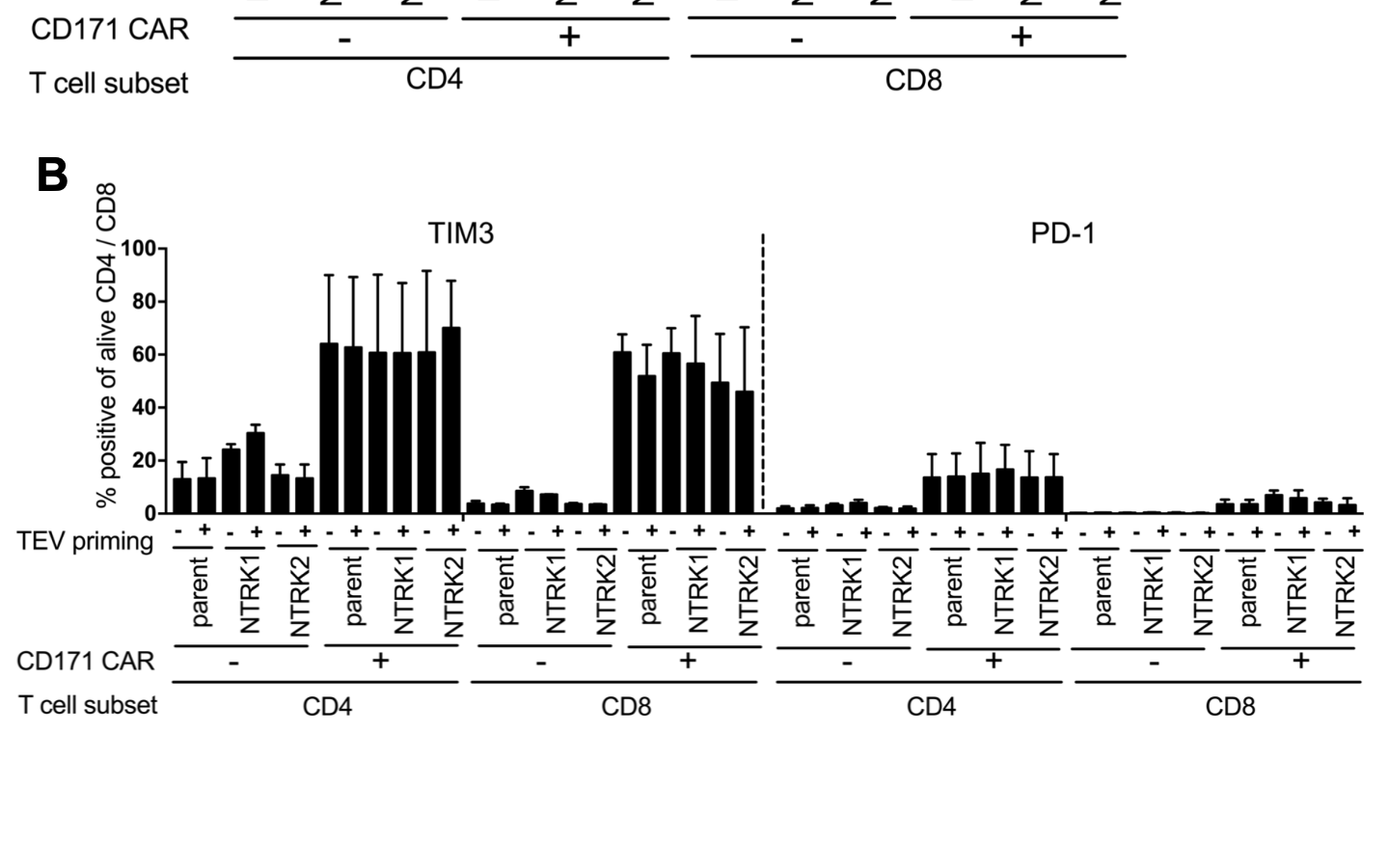
 Supplementary Figure 2. Prior TEV priming had no effect on expression of T cell activation or exhaustion markers. (A) Expression of activation marker CD137 and (B) exhaustion markers TIM3 and PD-1 on TEV-primed and unprimed CAR T cells after 24h tumor co-culture at an effector:target ratio of 1:2. Expression surface activation or exhaustion markers was determined via flow-cytometric analysis and depicted are CAR T cells double positive for CD4^+^ or CD8^+^ and activation or exhaustion markers. All cells were gated on single living cells. Depicted is mean of biological triplicates with SD as error bars.

## Supplementary Figure 3. SH-SY5Y-NTRK2 cells transduced to express high levels of CD171. CD171 cell surface expression on CD171-transduced and untransduced SH-SY5Y-NTRK2 cells detected with antihuman fluorochrome-conjugated CD171 antibody.

##

##
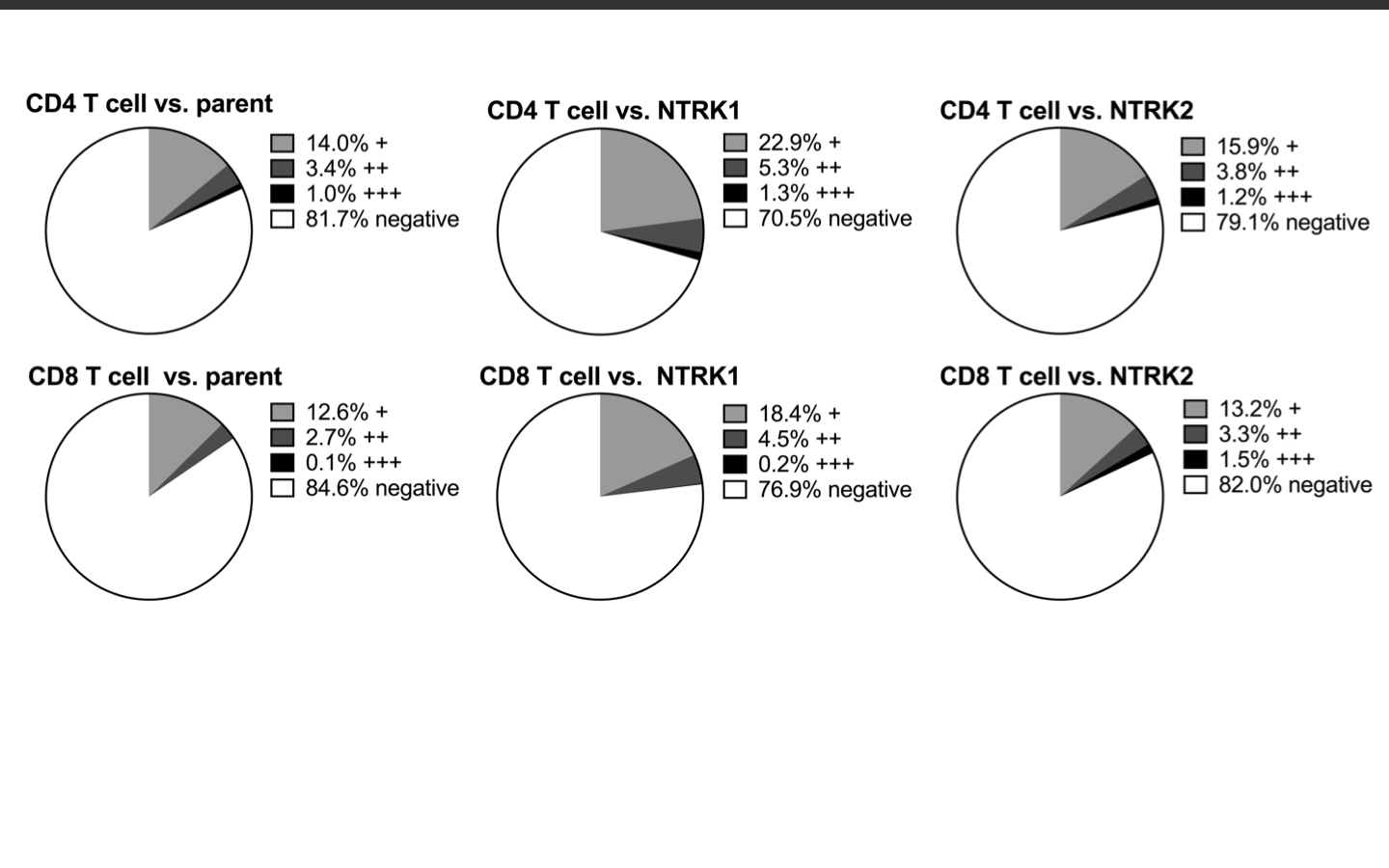


## Supplementary Figure 4. Distribution of no, single (+), double (++) or triple (+++) expression of exhaustion markers on CD4^+^ and CD8^+^ cells without CAR construct after 24h tumor co-culture in an effector:target ratio of 1:1. Single positivity (+) was defined as the sum of TIM3^+^ / PD-1^-^ / LAG3^-^, TIM3^-^ / PD-1^+^ / LAG3^-^ populations and TIM3^-^ / PD-1^-^ / LAG3^+^ populations. Double positivity (++) was defined as the sum of TIM3^+^ / PD-1^-^ / LAG3^+^ populations, TIM3^+^ / PD-1^+^ / LAG3^-^ populations and TIM3^-^ / PD-1^+^ / LAG3^+^ populations. Triple positivity (+++) was defined as TIM3^+^ / PD-1^+^ / LAG3^+^ populations. Negative expression was defined as TIM3^-^ / PD-1^-^ / LAG3^-^ populations.
